# Zika Virus Knowledge among Pregnant Women Who Were in Areas with Active Transmission

**DOI:** 10.3201/eid2301.161614

**Published:** 2017-01

**Authors:** Kate Whittemore, Anna Tate, Alex Illescas, Alhaji Saffa, Austin Collins, Jay K. Varma, Neil M. Vora

**Affiliations:** New York City Department of Health and Mental Hygiene, New York, New York, USA (K. Whittemore, A. Tate, A. Illescas, A. Saffa, A. Collins, J.K. Varma, N.M. Vora);; Centers for Disease Control and Prevention, Atlanta, Georgia, USA (J.K. Varma, N.M. Vora)

**Keywords:** Zika virus, viruses, Zika virus infection, travel, knowledge, practices, pregnant women, New York, United States, vector-borne infections

## Abstract

We surveyed women in New York, New York, USA, who were in areas with active Zika virus transmission while pregnant. Of 99 women who were US residents, 30 were unaware of the government travel advisory to areas with active Zika virus transmission while pregnant, and 37 were unaware of their pregnancies during travel.

Zika virus is primarily transmitted by the bite of infected *Aedes* mosquitoes; the virus can also cross the placenta of infected pregnant women, potentially leading to congenital infection and serious birth defects (*1*–*3*). As of October 7, 2016, a total of 617 cases of Zika virus infection had been identified among New York City (NYC) residents, including 72 cases among pregnant women (*4*).

Despite government advisories in place since early 2016 recommending that pregnant women avoid travel to areas with active Zika virus transmission (*4**,**5*), the NYC Department of Health and Mental Hygiene (DOHMH) saw an increase in weekly Zika virus test requests through the summer for women who had been in such areas while pregnant. This increase alerted the DOHMH to the need for additional messaging. To guide this communication, we conducted telephone surveys to evaluate Zika virus knowledge and practices among women in NYC who had been in such areas while pregnant.

In brief, during June 1–July 15, 2016, the DOHMH Zika Testing Call Center facilitated testing for 1,086 women >18 years of age because they were pregnant while in an area with active Zika virus transmission (*6*) (Technical Appendix). At the time of receiving the Zika virus test request, DOHMH collected demographic data, contact information and other pertinent clinical history on the patients; these 1,086 women were potentially eligible for the survey if their telephone number had been provided. The women were called in random order until ≈100 provided consent and completed the survey. Descriptive statistics were calculated for responses to each survey question. 

After 642 eligible women had been called, the target number of respondents had provided consent and completed the survey (n = 121; 18.8%); 67 (55.4%) respondents were interviewed in Spanish. We found no statistically significant differences in demographic characteristics between respondents and nonrespondents (Technical Appendix Table).

Of the 121 respondents, 99 (81.8%) were US residents (considered the United States their home). Approximately one third of the US residents (n = 30; 30.6%) were unaware of the government advisory (recommending that pregnant women avoid travel to areas with active Zika virus transmission) at the time of travel (Table). Nearly half (n = 43; 44.3%) did not know that there was active Zika virus transmission in areas where they traveled, and more than one third (n = 37; 38.5%) did not know that they were pregnant during travel. Of the 30 US residents who were aware of the government advisory, were aware of active Zika virus transmission in areas where they traveled, and knew that they were pregnant during travel, 7 (23.3%) still traveled because their trips were too expensive to cancel. Of 6 US residents who did not know about the government advisory but did know of active Zika virus transmission in areas where they traveled and did know that they were pregnant during travel, 5 (83.3%) said they would not have traveled had they known about the government advisory. The most frequently reported reason for travel among US residents was to visit friends or relatives (n = 68; 70.1%). 

**Table:** Knowledge about Zika virus infection among US residents who were pregnant at time of travel to areas with active Zika virus transmission, New York, NY, USA, June 1–July 15, 2016*

Characteristic	Total responses	Yes (%)*	No (%)*
Aware of government travel advisory at time of travel to areas with active Zika virus transmission	98	68 (69.4)	30 (30.6)
Aware that areas of travel had active Zika virus transmission	97	54 (55.7)	43 (44.3)
Aware of pregnancy status at time of travel to areas with active Zika virus transmission	96	59 (61.5)	37 (38.5)
Reason for travel			
Visiting friends or relatives	97	68 (70.1)	29 (29.9)
Tourism	97	52 (53.6)	45 (46.4)
Other	87	24 (27.6)	63 (72.4)
Business	97	5 (5.1)	92 (94.9)
Education	97	5 (5.1)	92 (94.9)
Service-related	97	4 (4.1)	93 (95.9)

*Column percentages do not total 100% because categories are not mutually exclusive. Denominator includes only those respondents who answered the question.

Among the women we surveyed, many were unaware of the government travel advisory, unaware of active Zika virus transmission in areas where they traveled, or unaware of their pregnancy during travel. However, our survey had limitations. The small sample size limited our ability to perform sophisticated analyses, and the potential for social desirability and recall bias are inherent to the study design. The survey questionnaire was not a validated instrument. Also, the women described here completed the survey after Zika virus testing; therefore, it is possible that they had a better understanding than the general public. 

Most participants in our survey were interviewed in Spanish. This finding underscores the need for providing educational materials in multiple languages. 

Although our findings cannot be generalized, they provide insight for increased and improved public health messaging. Public health authorities in the United States should continue to raise awareness among women of reproductive age about the risk for Zika virus infection from travel, enabling them to better make informed decisions. Women who are trying to become pregnant or who are pregnant should avoid travel to areas with active Zika virus transmission and, if they must travel, should talk to their healthcare provider first and take steps to minimize exposure to Zika virus. Furthermore, women who are trying to become pregnant should follow Centers for Disease Control and Prevention (Atlanta, GA, USA) guidelines on how long to wait to get pregnant after a potential Zika virus exposure (*7*). Women who want to avoid pregnancy and their male partners should use effective birth control correctly and consistently (*8*). Healthcare providers in the United States caring for pregnant women and women who are trying to become pregnant should routinely discuss travel history and travel plans with their patients.

Technical Appendix Additional materials and methods for survey of knowledge about Zika virus infection among pregnant women who were in areas with active virus transmission.

## References

[1] Centers for Disease Control and Prevention. Zika virus [cited 2016 Jul 20]. http://www.cdc.gov/zika/about/index.html

[2] Rasmussen SA, Jamieson DJ, Honein MA, Petersen LR. Zika virus and birth defects—reviewing the evidence for causality. N Engl J Med. 2016;374:1981–7. 10.1056/NEJMsr1604338 27074377

[3] Oduyebo T, Igbinosa I, Petersen EE, Polen KN, Pillai SK, Ailes EC, et al. Update: interim guidance for health care providers caring for pregnant women with possible Zika virus exposure—United States, July 2016. MMWR Morb Mortal Wkly Rep. 2016;65:739–44. 10.15585/mmwr.mm6529e1 27467820

[4] New York City Department of Health and Mental Hygiene. Zika virus [cited 2016 Oct 20]. https://www1.nyc.gov/site/doh/health/health-topics/zika-virus.page

[5] Centers for Disease Control and Prevention. Zika travel information [cited 2016 Jul 20]. http://wwwnc.cdc.gov/travel/page/zika-travel-information

[6] Lee CT, Vora NM, Bajwa W, Boyd L, Harper S, Kass D, et al.; NYC Zika Response Team. Zika virus surveillance and preparedness—New York City, 2015–2016. MMWR Morb Mortal Wkly Rep. 2016;65:629–35. 10.15585/mmwr.mm6524e3 27337505

[7] Centers for Disease Control and Prevention. Women trying to become pregnant [cited 2016 Jul 20]. http://www.cdc.gov/zika/pregnancy/women-and-their-partners.html

[8] Centers for Disease Control and Prevention. Women of reproductive age [cited 2016 Jul 20]. http://www.cdc.gov/zika/hc-providers/contraception.html.

