# Supplementary material for: Zika Virus Knowledge among Pregnant Women Who Were in Areas with Active Transmission
Source: Emerg Infect Dis. 2017 Jan;23(1):164–6. doi: 10.3201/eid2301.161614 (PMC5176227; doi:10.3201/eid2301.161614)
Supplement: Technical Appendix — Additional materials and methods for survey of knowledge about Zika virus infection among pregnant women who were in areas with active virus transmission. [file 16-1614-Techapp-s1.pdf]

Article DOI: http://dx.doi.org/10.3201/eid2301.161614

Zika Virus Knowledge among Pregnant Women Who Were In Areas with Active Transmission

Technical Appendix

Methods

During June 1–July 15, 2016, the NYC Department of Health and Mental Hygiene (DOHMH) Zika Testing Call Center facilitated testing for 1,086 women ≥18 years of age because they were pregnant while in an area with active Zika virus transmission (*1*). Women were eligible for the survey if they had a telephone number on record and their healthcare provider had an email address on record (n = 943; 86.8%). Women's healthcare providers were notified about the survey by email 7 days before conducting the survey; one provider declined her patient's participation. The remaining 942 women's names were randomly ordered, and the women were called until a target of ≈100 had provided consent and completed the survey. Because the survey was part of the ongoing DOHMH emergency public health response to Zika virus, the target of ≈100 women was chosen arbitrarily.

Only 1 call attempt was made per eligible woman. Surveys were conducted in English and Spanish (or by an interpreter for 1 Mandarin-speaking woman). All survey respondents were asked about their general knowledge of Zika virus; those who knew at the beginning of the survey that "Zika" is an illness were asked questions about preventive measures taken. Women who reported that they considered the United States their home ("US residents") were asked additional questions related to their knowledge of the government travel advisory for pregnant women at the time of travel, details about their travel, and knowledge of their pregnancy status during travel.

Descriptive statistics were calculated for responses to each survey question; except where noted, denominators for frequencies reflect the number of responses for that question and do not include missing responses (missing responses occurred either because a respondent was not

asked a question based on the response to a previous question or because the respondent declined to answer the question). A 1-way analysis of variance was used to test for differences between means in age among respondents, women who did not consent, and all eligible women; a chi-square test was used to test for differences in race/ethnicity among the groups. This survey was part of the ongoing DOHMH emergency public health response to Zika virus and was determined by DOHMH and CDC to be a non-research activity.

Results

Of 642 (68.2%) women called, 407 (63.4%) did not answer the phone, 114 (17.8%) refused consent to be interviewed, and 121 (18.8%) consented. Most respondents identified as Hispanic (n = 78; 68.4%) and 24 (21.1%) identified as non-Hispanic Black. There were no statistically significant differences in demographic characteristics between respondents and either women who did not consent to participate or all eligible women. (online Technical Appendix Table 1)

Among those who answered questions on knowledge of modes of Zika virus transmission, 112 (100.0%) knew that Zika virus is transmitted by mosquito bite, 106 (93.0%) through sexual contact, and 111 (98.2%) from mother-to-fetus; 100 (89.3%) knew that there is a link between Zika virus infection during pregnancy and birth defects. However, 70 (63.1%) respondents incorrectly believed that most persons with Zika virus infection have symptoms. Among the 67 respondents who knew at the beginning of the survey that “Zika” is an illness, >25% reported not practicing each of the preventive actions for Zika virus infection that we separately evaluated. (online Technical Appendix Table 2) More than one third (n = 32; 34.4%) of US residents reported visiting a healthcare provider for advice before traveling.

Reference

1. Lee CT, Vora NM, Bajwa W, Boyd L, Harper S, Kass D; NYC Zika Response Team. Zika virus surveillance and preparedness—New York City, 2015–2016. MMWR Morb Mortal Wkly Rep. 2016;65:629–35. PubMed http://dx.doi.org/10.15585/mmwr.mm6524e3

Technical Appendix Table 1. Demographics of study population, New York, New York, USA, June 1–July 15, 2016

Characteristic	Respondents*	Women who did not consent*	All eligible women*	p value
Total, No.	121	114	943	
Age, years	29.1	29.3	28.9	0.68
Race/Ethnicity, No. (%)				0.09
Hispanic	78 (64.5)	61 (53.5)	516 (54.7)	
Non-Hispanic Black	24 (19.8)	26 (22.8)	243 (25.8)	
Non-Hispanic White	4 (3.3)	10 (8.8)	61 (6.5)	
Non-Hispanic Asian	5 (4.1)	1 (0.9)	21 (2.2)	
Non-Hispanic American Indian/Alaskan Native	1 (0.8)	4 (3.5)	10 (1.1)	
Non-Hispanic Other	2 (1.7)	4 (3.5)	12 (1.3)	
Missing	7 (5.8)	8 (7.0)	80 (8.5)	

*Denominator includes missing values

Technical Appendix Table 2. Actions taken to prevent Zika virus infection by women who were in areas with active Zika virus transmission while pregnant, New York, New York, USA, June 1–July 15, 2016*

Preventive action	Total responses	Yes (%)†‡	No (%)†‡
Used insect repellent most of the time while outside	67	47 (70.1)	20 (29.9)
Wore long sleeves and pants most of the time while outside	67	36 (53.7)	31 (46.3)
Covered feet most of the time while outside	67	42 (62.7)	25 (37.3)
Treated clothing with insect repellant	67	23 (34.3)	44 (65.6)
Used window screens	67	42 (62.7)	25 (37.3)
Used air conditioning most of the time‡	66	39 (59.1)	27 (40.9)
Covered and removed containers of standing water	67	46 (68.7)	21 (31.3)
Used mosquito nets most of the time while sleeping	67	22 (32.8)	45 (67.2)

*Survey respondents received this series of questions only if they knew at the beginning of the survey that "Zika" is an illness.
†Column percentages do not total 100% because categories are not mutually exclusive.
‡Denominator includes only those respondents who answered the question.

Original Questionnaire

Date (MM/DD/YYY) _____________________ Patient Access ID Number __ __ __ __
Name of Interviewer _____________________

Zika Phone Survey Questions

OPERATOR INSTRUCTIONS: Read each question and ALL of the corresponding answer choices out loud. After you have read the entire question and answer choices, THEN ask the participant what answer choice they would like to choose (try to not let the participant answer the question until they've heard all answer choices). Enter the participant's answers in the database.

******NOTE: If a patient asks a CLINICAL QUESTION, direct them to follow up with their PHYSICIAN and do NOT attempt to answer the question.***

[Attempt to call the patient 1 time. If the patient does not answer the phone, leave the following message]:

"Hello, this is a survey call. There is no need to call us back. Thank you and have a nice day."

[OPERATOR SCRIPT – INVITATION TO PARTICIPATE]:

"Hello, this is ___________ from the New York City Department of Health. May I please speak to ___________ [*patient's name*]? [*Once you confirm you're speaking to patient*] **We are conducting a survey to learn more about your knowledge and experience with Zika. You are receiving this call because your healthcare provider ordered Zika testing for you through the Health Department. Please keep in mind that we are *only* calling in regards to our survey, and we do *not* have any information about your test results. The survey should**

take about 10 to 15 minutes. You do not have to answer any question you do not want to answer.
If you choose to participate, there are no gifts or rewards, and there are no risks. Other pregnant women may benefit from your participation. .
Are you willing to participate in the survey over the phone?"
[*OPERATOR SCRIPT*]:
[IF YES]: Thank you for agreeing to participate.
Before we start the survey, it is important to understand that Zika is spreading in the Caribbean, Central America, and parts of South America. Zika is not currently spreading in New York City. [*Proceed to Question 1.*]
[IF NO]: Okay, no problem. If you have additional questions about Zika, you can visit the New York City Department of Health website for more information: www.nyc.gov/health/zika. Thank you for your time and have a nice day.

1. **What is Zika?** *Choose one.* [*If participant does not answer correctly, read the first sentence of the "WHAT IS ZIKA" script on the interviewer information sheet and skip to Question 5.*]
    a. A mosquito
    b. An illness
    c. A country
    d. Don't know
2. **When was the first time you ever heard of Zika?** *Choose one.*
    a. Last year or before last year
    b. A few months ago
    c. A few weeks ago
    d. A few days ago
3. **How did you first hear about Zika?** *Choose one.*
    a. TV news
    b. TV advertisement
    c. Online search
    d. Online advertisement
    e. Radio
    f. Family or friends
    g. Healthcare provider
    h. Pharmacy
    i. Subway ads
    j. Other ______________
4. **Your doctor told us that you were in an area in which Zika was spreading while you were pregnant. For example, you may have been in Central America, South America, or the Caribbean while pregnant. Next, I will ask you a series of questions about your activities while you were in that area in which Zika was spreading. Please answer 'yes' or 'no'.** *Choose one for each question.*
    a. While you were in that area, did you use insect repellent most of the time you were outside? Y N
    b. While you were in that area, did you wear long sleeves and pants most of the time you
    were outside? Y N

c. While you were in that area, did you cover your feet most of the time you were outside?
Y N
d. While you were in that area, did you treat most of your clothing with insect repellant? Y N
e. While you were in that area, were there window screens in the place you stayed? Y N
f. While you were in that area, did you use air conditioning most of the time in the place you stayed? Y N
g. While you were in that area, did you or whomever you were staying with cover and remove containers of standing water? Y N
h. While you were in that area, did you use mosquito nets most of the time you slept? Y N

5. **Do you consider the United States to be your home?** *Choose one.* [*If no, skip to Question 11.*]
    a. Yes
    b. No
6. **Before you traveled to that area in which Zika is spreading, did you know that the government advises against going to an area where Zika is spreading while pregnant?** *Choose one.*
    a. Yes
    b. No
7. **At that time you were in that area, did you know that Zika was spreading in that area?** *Choose one.*
    a. Yes
    b. No
8. **At that time you were in that area, did you know that you were pregnant?** *Choose one.*
    a. Yes
    b. No
9. **[*IF YES to 6, 7, and 8*]: Did you go because your trip was too expensive to cancel?** *Choose one.*
    A. Yes
    B. No
10. **[*IF NO to 6, YES to 7 and 8*]: If you had known about the government warning, would you have still gone to an area where Zika is spreading?** *Choose one.*
    a. Yes
    b. No
    c. Don't know
11. **Next, I'm going to ask you a series of questions about why you traveled to the area where Zika is spreading.** *Choose one for each question.*
    a. Did you go for business? Y N
    b. Did you go for education? Y N
    c. Did you go for service? Y N
    d. Did you go for tourism? Y N
    e. Did you go to visit friends or relatives? Y N

f. Other_____________________________ Y N

12. **Did you visit a healthcare provider in the United States before you went to this area where Zika is spreading to get advice on how to stay healthy while traveling?** *Choose one.*
    a. Yes
    b. No
13. **Next, I will ask you a series of questions about how Zika is transmitted. Please answer 'yes' or 'no'.** *Choose one for each question.*
    a. Can Zika be transmitted by coughing? Y N
    b. Can Zika be transmitted by sneezing? Y N
    c. Can Zika be transmitted by having sex with someone who has Zika? Y N
    d. Can Zika be transmitted by the bite of an infected mosquito? Y N
    e. Can Zika be transmitted by shaking hands with someone who has Zika? Y N
    f. Can Zika be transmitted from an infected pregnant woman to her baby? Y N
14. **Do most people with Zika have symptoms?** *Choose one.*
    a. Yes
    b. No
15. **Do you think there is a link between Zika during pregnancy and birth defects?** *Choose one.*
    a. Yes
    b. No
16. **Do you agree with the following statement: All pregnant women who while pregnant were in an area where Zika is spreading should be tested for Zika.** *Choose one.*
    a. Strongly agree
    b. Agree
    c. Neutral
    d. Disagree
    e. Strongly disagree
17. **At this time, do you feel like you have enough information about Zika?** *Choose one.*
    a. Yes
    b. No
        i. [*IF NO*]: What additional information would be helpful to you?
18. **We're interested in raising more awareness about the risk of Zika in women who travel to Latin America or the Caribbean. Would you be willing to have someone from our communications office speak to you about Zika? If you agree, a Department of Health employee may call you to ask some questions and possibly arrange an interview.** *Choose one.*
    a. Yes
    b. No
19. **How could the Department of Health improve how we spread information about Zika?**
20. **Is there anything else you would like to tell us about your experience with Zika testing in New York City or anything else related to Zika?**

Thank you. If you have additional questions about Zika, you can visit the New York City Department of Health website for more information: www.nyc.gov/health/zika. Thanks again for your time – we really appreciate your insight. Have a nice day.
